# Progress of Dental Surgery in the United States

**Published:** 1880-11

**Authors:** J. B. Patrick

**Affiliations:** Charleston, President of the National Dental Association, before the Recent Meeting in New York.


### ARTICLE II.

### *Progress of Dental Surgery in the United States.*

**Opening Address of Dr. J. B. Patrick, of Charleston, President of the National Dental Association, before the Recent Meeting in New York.**

*Gentlemen of this United Convention.*—I congratulate you that we have been permitted to assemble once more in a representative capacity for the purpose, not only of interchanging thought concerning the progress of our profession, but of renewing the pleasant social relations that have proved such a bond of union between many of us in the past. In the interval that has occurred since the members of the respective associations now present met together, changes have undoubtedly taken place in our respective homes for better or worse, as the case may be, but here we meet in common to take note of those changes only that have influenced our several societies, and to put the stamp of our fresh initials on the milestones that mark the progress of our science.

It is a source of gratification likewise to observe the spirit of unity and cordiality with which the Convention has assembled. Coming as we do from remote portions of our common country, some from its villages and towns, and others from its great cities, where abound mighty opportunities for observation and improvement, I trust that all of us have brought hither more or less of the results of our experience, and are prepared to contribute the same to that which at the end of our deliberations may be regarded as the voice and intellectual outpour of this body.

Of the importance of such a national organization as we contemplate there can be but one opinion. Without interfering with the lines of demarcation that surround the local societies, or seeking to disturb whatever of good may emanate from their existence, our present effort should be to consolidate all interests that are mutual, and stimulate all purposes that have a common object. Of jealousies among us, thank God, there are none; but of high aspirations there are a multitude, and whether we come from the Dental Society of the State of New York, the American Dental Convention, the American Dental Association or the Southern Dental association, it is that we may stand shoulder to shoulder in achieving the grandest results that may be commanded by unity of thought. In the promulgation of this thought we shall find our main strength, for it will flow from a centre whose interwoven fibres are in themselves strong, and whose forces need only to be distributed through the arteries of one homogeneous body to attain all the possible benefit that can enrich our profession. Permit me to say that this I regard as one of the largest elements of our success, for while each State may in time possess its local society—and the more the better—each will contribute of its membership to the grand national association that we have in prospect, and thus through attrition enlarge the field of our experience, and supply from many sources food for our instruction.

The necessity for such a concentration of useful influences must be apparent to even a superficial observer. If we scrutinize the records we find scattered through them information of no inconsiderable value, and yet it might be made doubly so if it could find voice through a national vehicle, instead of being scattered like oases in the broad Sahara of dental literature. Do not let me be misunderstood. I bow my knee in tribute to the noble army of authors and essayists in our profession who have done and are doing so much to disseminate knowledge, but you will concede that where the gleanings are so numerous and the scope of discovery is so wide, there is a greater need than ever, that as a community interested in every new fact bearing on our science, we should be enabled to avail ourselves of every step in advance; and this, gentlemen, is one of the advantages that will accrue from our national organization.

The age in which we live everywhere witnesses the crystallization of ideas, and their march through given channels. The generation is eminently practical and utilitarian. We are creating as much as we are improving. Each speciality has its Columbus, and each Columbus is making his voyage of discovery, and on it is thrown a camera that exhibits in the brightest light the progress and development of the art or science to which that specialty belongs. Scan the field of medicine, anatomy and physiology, and recall the many departments into which it has been subdivided, and on which are at work some of the grandest intellects. Mark the labors of the engineer, the bridge, the railway and tunnel builders, the men who are making steam and electricity handmaids of the people's growth, and leaving an impress deeper than ever was cut before in the history of mankind. See Edison in his laboratory patiently revolving problems for supplying light and giving life to machinery through a new motor. Note the little vessel that has just crossed the Atlantic to test the practicability of condensing and reusing its own steam, and economizing by many per cent. the use of fuel, thus annually saving millions of dollars. See chemistry simplifying the arts that they may be domesticated. Observe the perfection of travel on your elevated railroads, the bridging of the East River, the tunneling of the Hudson, the opening of the Suez Canal, the boring of the Alps between Italy and France, in short, turning the eye on every side and where in a similar period of the world's history have grander results been achieved?

And what is true of other departments of science is equally true of our own. Our brainworkers have not been idle, and our tablets are scored all over with the intellectual victories that have been won by the members of the dental profession in their encounters with the inexperienced that characterized to a considerable extent its incipiency as a profession less than forty years ago.

It would require more time than may be permitted in a brief introductory address like the present to review the changes and improvements that have taken place among us, even during a quarter of a century. There are those here who remember how the tooth puller, the barber and the country peddler went almost hand in hand, and there was no thought that we should live to see the delicate and effective appliances now in use for preserving and perpetuating the teeth and removing the evils of hereditary growth. Art has, however, changed the then existing conditions. The freaks of nature have come within the domains of scientific mechanism, and the degeneration caused by luxury or vice is obedient in its yielding to the exact law through which we enforce a return to Nature and the designs of Nature's God.

While a few years ago the dentist was regarded as little better than a mere artisan, whose knowledge did not extend beyond his simple tools of trade, to-day finds him recognized as the member of an honorable profession, interlinked with every other connected with the science of Anatomy and Physiology, and a contributor to and an important adjunct of the art of healing and reproduction. It has been well said that it requires greater professional skill "properly to handle the teeth than it does to set an arm or extract an eye." This may seem to be a broad assertion, but there are many among the present constituency who have at various times in their experience been brought face to face with cases in which the nicest judgment of the surgeon, the most exhaustive skill of the anatomist, and the keenest touch of the operator were required to produce the effect desired.

It has been estimated that the 12,000 dentists in the United States annually pack into the teeth of the people not less than half a ton of pure gold, costing half a million of dollars, besides about four times as much cheaper material, such as silver and platinum, worth $100,000 more. It is also said that there are made every year about 3,000,000 artificial teeth mounted on gold, platina, rubber, or other materials. These facts, if correct, will convey a partial idea of the activity of our profession, and yet it may be safely asserted that not more than one person in twenty has perfect teeth. Such a vast dentile must naturally suggest that we cannot remain idle or unmindful of opportunities. We have to study complex conditions without number, and to avail ourselves of every discovery in other arts that can possibly affect our own. For years chemists and others have been engaged in voyages of exploration to discover something that shall more perfectly harmonize with the dentos than the metals which we use at present, without the present drain upon the operator and the patient, but we have progressed only in so far as to learn the extent of our deficiencies. I need not further dwell on this point. Time, experience and the laboratory must give us the solution of the question that is involved in this department of our science. That some substance will eventually be accepted by the profession as an equivalent of the best now in use, and superior to all others yet in use, as regards durability and affiliative qualities, I have no doubt. Meanwhile we can only be patient in utilizing the materials at hand and noting the experiments that are from time to time reported by our co-laborers in the field.

In this connection it is gratifying to record other results as the outgrowth of the improvement that has taken place in our profession during the last quarter of a century. Admitting all the advantage that has accrued from desirable and effective mechanism and the use of instruments that a few years ago were unknown, it is the universal testimony that dentists nowadays operate with greater delicacy of touch, affording less inconvenience to the patient, and leaving behind fewer of the after traces of treatment that were observable in former years. I do not stop to question that this is an important point gained, for it is answered by the fact that we possess greater facilities than before, even though they may not be accompanied by a superior intelligence or an experience any more diversified. Manipulative skill may be said to have become subjective to machinery.

Again, the suggestive instructions of the collegiate course have more or less exercised a predominating influence upon the profession. Some among the students have found occupation for their talent in the perfection of artificial teeth; others have made a specialty of contrivances for the correction of irregularities and the removal of deformities; many have devoted themselves simply to the art of filling and making perfect the teeth, while a few animated by a noble ambition have sought to cover all these aims and be prepared beside to stand in the forefront of their profession, ready to respond to the grandest requirements of the science, and with their knowledge of constitutional or theoretical treatment, when needs be, aid their professional brethren in other departments of the healing art.

In no sense, therefore, has our profession deteriorated. We stand before the public a compact, solid mass of educated gentlemen—the servants and the confidants of that public—and intent only on the alleviation of the suffering that is the common lot of humanity. While we are moving forward with all consistent speed, availing ourselves of all the factors that make the world's sum of progress, we are content to be measured by the modest achievements that come strictly within the purview of our life's work. Our duty, however, does not alone consist in meeting our adversary, disease, half way; he must be met at the threshold.

Brought as we are into daily communication with father, mother and children, frequently covering more than a generation of growth, it is possible for us, both as individuals and as an organization, to do this, to educate the masses and to teach them somewhat of that harmony of life that is demanded by physiological law and the inevitable lesson that follows disobedience. I have no hesitation in saying that the next generation will witness a greater abnormality in dental development than we have yet seen. It is for this simple reason that there is a great tendency to nervous and cerebral diseases, resulting from fast living and injudicious food. It shows itself particularly in the precocity of children. It is in my experience that in a majority of cases your precocious child has a mouth full of fangs, and a dental organization throughout that is hereditarily bad.

To us, gentlemen, belongs the task of interposing our skill as a barrier to the encroachment of these and all other forms of dental disease. In conclusion, gentlemen, I have but a few more words to utter. Our Convention promises to be an important one, the most important events indeed in the history of the profession. You are in person the embodiment of the growth of that profession, and you have reflected honour on its shield. Science in your hands has suffered no corruption nor has been permitted to stagnate.

While there may have been a long interval between the seed and the timber, we have already seen the bud and flower and fruit; we are progressing step by step toward still higher planes, with the consolation of knowing that what is gained by one man is invested in all men, and is a permanent investment for all time. Again felicitating on the occasion that has brought so many of our number together, and expressing the hope that it will be productive of the pleasantest memories, I announce this Convention ready for the transaction of business.

---

